# The Impact of the COVID-19 Pandemic on Pregnant Women with Perinatal Anxiety Symptoms in Pakistan: A Qualitative Study

**DOI:** 10.3390/ijerph18168237

**Published:** 2021-08-04

**Authors:** Nida Rauf, Shaffaq Zulfiqar, Sidra Mumtaz, Hadia Maryam, Rabail Shoukat, Abid Malik, Armaan A. Rowther, Atif Rahman, Pamela J. Surkan, Najia Atif

**Affiliations:** 1 Human Development Research Foundation, House No 06, Street No 55, F-7/4, Islamabad 44000, Pakistan; nida@hdrfoundation.org (N.R.); shaffaq.zulfiqar@hdrfoundation.org (S.Z.); sidra.mumtaz@hdrfoundation.org (S.M.); hadia.maryam@hdrfoundation.org (H.M.); rabail.shouket@hdrfoundation.org (R.S.); abid.malik@hdrfoundation.org (A.M.); 2 Department of Research, Rawalpindi Medical University, Tipu Rd, Chamanzar Colony, Rawalpindi 46000, Pakistan; 3 Department of International Health, Johns Hopkins Bloomberg School of Public Health 615 N. Wolfe St., Baltimore, MD 21205, USA; armaan.rowther@jhu.edu (A.A.R.); psurkan@jhu.edu (P.J.S.); 4 Institute of Population Health, University of Liverpool, Liverpool L12 2AP, UK; atif.rahman@liverpool.ac.uk

**Keywords:** COVID-19, pregnant women, anxiety symptoms, mental health, low- and middle-income countries

## Abstract

The impact of coronavirus disease 2019 (COVID-19) on people with existing mental health conditions is likely to be high. We explored the consequences of the pandemic on women of lower socioeconomic status with prenatal anxiety symptoms living in urban Rawalpindi, Pakistan. This qualitative study was embedded within an ongoing randomized controlled trial of psychosocial intervention for prenatal anxiety at a public hospital in Rawalpindi. The participants were women with symptoms of anxiety who had received or were receiving the intervention. In total, 27 interviews were conducted; 13 women were in their third trimester of pregnancy, and 14 were in their postnatal period. The data were collected through in-depth interviews and analyzed using framework analysis. Key findings were that during the pandemic, women experienced increased perinatal anxiety that was linked to greater financial problems, uncertainties over availability of appropriate obstetric healthcare, and a lack of trust in health professionals. Women experienced increased levels of fear for their own and their baby’s health and safety, especially due to fear of infection. COVID-19 appears to have contributed to symptoms of anxiety in women already predisposed to anxiety in the prenatal period. Efforts to address women’s heightened anxiety due to the pandemic are likely to have public health benefits.

## 1. Introduction

The World Health Organization (WHO) declared coronavirus disease 2019 (COVID-19) a public health emergency of international concern [1]. Recent studies suggest that the pandemic has not only affected patients’ physical health, but also had a profound psychological impact on the population [2], resulting in increased emotional distress and psychological illness [3,4]. A systematic review of 19 studies on the effects of COVID-19 on psychological outcomes in the general population reported an association with highly significant levels of psychological distress that, in many cases, would meet the threshold for clinical relevance [5]. 

Pandemics have an even greater psychosocial effect on vulnerable populations such as people experiencing poverty [6], those with physical and mental illnesses [7], and pregnant women [8]. A US survey of 2740 pregnant women during the COVID-19 pandemic indicated that the pandemic significantly impacted participants’ mental health and increased pregnancy-specific anxiety [9]. Similar results originating from other countries such as Canada [10,11], Iran [12], and China [13] also demonstrated an upsurge of anxiety symptoms in pregnant women. These studies suggest that COVID-19 is a risk factor for perinatal anxiety [14]. Higher symptoms of anxiety and depression in pregnant women are associated with strained relationships and social isolation. These symptoms are also related to increased concerns about the lack of essential prenatal care, concern about the effects of COVID-19 on the safety of mothers and babies [15], and effects on their obstetric decision-making [16]. 

The first COVID-19 case in Pakistan was reported in February 2020 [17]. Since then, the pandemic has continued to take a serious toll, severely affecting the country’s already burdened healthcare system and struggling economy. In Pakistan, pre-pandemic rates of anxiety were already higher than in most low- and middle-income countries [18], especially among women during pregnancy and the postnatal period [19]. Coupled with a lack of healthcare services [20] and a large treatment gap for mental illnesses [21], the current pandemic could have a profound impact on the well-being of women during the pre- and postnatal period.

The pandemic has prompted a number of surveys as well as clinical and epidemiological studies. However, there has been a dearth of qualitative research exploring the effects of the COVID-19 pandemic on experiences of anxious women in the pre- and postnatal period. This study aims to provide in-depth exploration of the perceptions, fears, and perceived impact of COVID-19 among women with preexisting anxiety symptoms in the late prenatal and postnatal periods in Pakistan. 

## 2. Materials and Methods

### 2.1. Settings and Participants

This study was embedded within the Happy Mother Healthy Baby (HMHB) randomized controlled trial, which aims to test the effectiveness of a psychosocial intervention for prenatal anxiety [22]. The participants in the trial were recruited from the Obstetrics/Gynecology Department of Holy Family Hospital, a tertiary care hospital at Rawalpindi Medical University in Rawalpindi, Pakistan. The inclusion criteria for the trial required women to have ≤ 22 gestational weeks of pregnancy, age ≥ 18 years, residence ≤ 20 km from Holy Family Hospital with the intent to reside in the area until the study’s completion, ability to understand spoken Urdu, and at least mild anxiety on the HADS anxiety subscale (score ≥ 8) [23] in the absence of a depression diagnosis according to the Structured Clinical Interview for DSM Disorders (SCID) [24,25]. The participants were purposively selected from the intervention arm based on their age, education, and number of children to allow maximum variation in the sample. These participants received their intervention between June 2020 and September 2020, during the COVID-19 pandemic. The COVID-19 pandemic reached Pakistan on 26 February 2020, and partial lockdown measures were implemented nationwide on 24 March 2020. During the pandemic, healthcare services were put on alert to sustain a balance between emergency, specialty, and outpatient services while keeping precautionary measures in place [26,27]. The nation’s first wave of COVID-19 cases began in late May 2020, peaked in mid-June, and ended in mid-July. Several restrictions were imposed in the study area during this period; these included the closure of hospital outpatient departments and educational institutions and a complete ban on intercity, inter-district, and interprovincial public transport as well as on large public or private gatherings [28].

### 2.2. Data Collection

The data of 27 women were collected through phone interviews. Prior to data collection, verbal explanation of the study was given over the phone to all the participants. The participants were informed of their right to withdraw from the study at any time. Those who gave consent were interviewed. In total, 29 women were approached, out of which 27 agreed to take part, with two women declining due to the lack of time.

An interview topic guide was developed, pilot-tested with two women, and revised based on the feedback received. In-depth interviews aimed to explore the participants’ (1) understanding of the COVID-19 pandemic, (2) most pressing concerns in relation to their pregnancy and newborn, (3) perceptions of the impact of COVID-19 on their emotional and physical well-being, and (4) experiences with healthcare during the pandemic. All the participants were given the choice to be interviewed at a time that was convenient for them. In any situation where privacy during the interview could be compromised, the participants were given the choice of stopping and rescheduling the interview.

The interviews were conducted by two research assistants with prior experience collecting qualitative data face-to-face. They received additional training regarding how to conduct in-depth interviews by phone. The training covered building rapport before the interview, ensuring confidentiality, sustaining participant attention during the interview, providing breaks if needed, and establishing mitigation strategies in case of a poor connection, a dropped call, or privacy being compromised. The interviewers were also trained to stop the interview upon hearing any comment by the participant indicating intent to harm herself or others and to do a risk assessment by asking three questions: (1) how frequently was the participant experiencing such thoughts; (2) had she made any plans to act upon these thoughts; and (3) if yes, did she have any means to do so. The interviewers then discussed the participant’s responses with a trained psychiatrist (A.M.) who took further actions according to the protocol (e.g., referral to a specialist facility). All the interviews lasted between 30 and 45 min and were audio-recorded. All the recorded data were transcribed and analyzed in Urdu by the qualitative research team. 

### 2.3. Data Analysis

The processes of data collection and analysis were carried out simultaneously using framework analysis. This approach was chosen because of its robust and systematic method of analyzing data that provides an audit trail of the main findings [29]. Data analysis utilized all the five steps of the framework analysis method: familiarization, coding, indexing, charting, and mapping and interpretation of the data [30]. Once the interview was transcribed, it was read and reread to identify emergent codes, subthemes, and themes. This led to the development of a thematic framework, in which each theme and subtheme was given an index number and applied to the raw data. The indexed sections of the raw data were placed in the thematic framework to create thematic charts. Finally, key elements of the charts were critically examined to understand links and associations that facilitated the interpretation of the data. To improve the rigor of the data analysis, each transcript was analyzed by two researchers. Then, the findings were discussed with the senior researcher (N.A.), and discrepancies were resolved while revisiting the raw data and referring to the field notes. Reflexivity was ensured by maintaining field notes and engaging in regular discussions to reflect upon the process of analyzing the data and assess any assumptions. This helped ensure that information was analyzed from the participants’ perspectives.

## 3. Results 

In total, 27 women were interviewed during their third trimester of pregnancy (*n* = 13) or in the postnatal period (*n* = 14). The majority of the participants were in their twenties or early thirties and had five or more years of schooling. Twenty-one women out of 27 lived in joint family systems (consisting of a married woman and her children living with the husband, his parents, and/or his siblings). Table 1 shows the participants’ characteristics.

**Figure 1:**
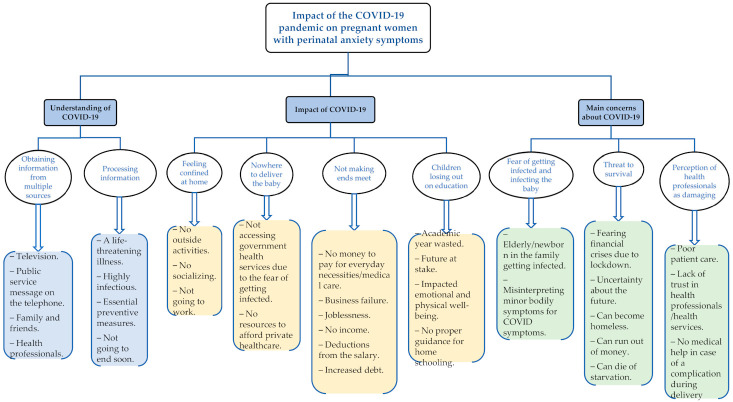
Emergent themes, subthemes, and codes from thematic analysis.

The data analysis generated three themes and nine subthemes (see Figure 1 below). These themes represent the participants’ understanding of the pandemic, its impact on them, and their worst fears related to it.

### 3.1. Understanding of the Pandemic

#### 3.1.1. Obtaining Information about COVID-19 from Multiple Sources

The majority of participants reported obtaining information about the pandemic through multiple sources, including family members, friends, health professionals, television programs, public service messages sent to phones, and the research therapists from whom they were receiving HMHB sessions. The information raised their awareness of the pandemic and helped them take precautionary measures. A 34-year-old woman, who received information on COVID-19 from television, reported, “Yes, my daughter and I are healthy due to the recommendations we used to hear from the television. I might have caught this virus if I had not followed those guidelines” (M08). Some participants, however, found the information from different sources to be inconsistent and confusing. For instance, one participant reported, “I heard that there was this lady who had bat soup somewhere due to which it [the virus] has spread” (M27). Another young expectant woman with low literacy stated, “There is a girl living near my mother’s home. She has this virus... A few people say it’s not real, others say it’s real. That’s why we stay at home most of the time” (M23).

#### 3.1.2. Processing Information on COVID-19

The data indicated that information the participants received about the pandemic helped the majority of them become more aware of the mechanisms of viral transmission and the importance of taking preventive measures. Furthermore, a campaign by the Pakistani Ministry of Health toward the start of the pandemic in March 2020 used the public service message “we must fight, not fear, the coronavirus.” Some of the participants reported not feeling afraid of the pandemic but rather taking necessary measures to stay safe from it, as stated by a young participant living in a joint family: “Precautions are a must. We don’t have to be afraid; we have to fight it by washing our hands frequently, wearing a mask and gloves when going outside. Also, [we should] sanitize and wash our hands upon returning” (M02).

In contrast, information about the possible effects of COVID-19 raised anxiety in some of the participants. A participant reported having no fear until she started hearing news and seeing people die on television: “I wasn’t aware of this virus before, that’s why I wasn’t afraid of it. We don’t go out of the house much. That is why I didn’t know much about it. But now I am worried by seeing corona patients on the television and hearing [that] so many people are dying from it” (M13). Another participant became preoccupied with thoughts of death after learning of the large numbers of people dying every day during the peak of the pandemic: “When I heard news on the television reporting the number of people who have died due to coronavirus and that this number was increasing every day, I was thinking about death all the time” (M08). Some of the participants refused to visit the hospital for antenatal checkups due to fears of getting infected. A pregnant woman stated, “I have heard it can make the person unwell, you can have a high fever and cough, and it can be passed on from an infected person to another person. It is best not to go to crowded places. That’s why I didn’t go to the hospital for my checkups. They say hospitals are coronavirus hubs” (M16).

### 3.2. Impact of the COVID-19 Pandemic

#### 3.2.1. Feeling Confined at Home

All the participants reported some impact of the COVID-19 pandemic on most aspects of their day-to-day lives. It created a major barrier to their activities outside the home such as shopping, visiting family and friends, and attending ceremonies such as weddings and funerals. For most participants, these activities had been helpful distractions from their strenuous daily routines and everyday worries. Not being able to engage in these activities contributed to increased anxiety. A participant recalled staying at home when she was pregnant, believing that she was at a higher risk of getting infected, and feeling confined: “I just stayed inside the house because I heard that pregnant women are at a higher risk of getting it. That’s why I did not step outside the house. At times I felt [like I was] restricted to these four walls and was doing nothing other than worrying” (M08). Another participant, living in a nuclear family, reported missing visits to her maternal home, which used to bring her much comfort and support: “I used to visit my mother’s home once a week. Now, due to this virus, I can only visit my mother’s home once a month” (M10). 

Most of the participants in the postnatal period were worried about their newborns getting infected with coronavirus and therefore preferred staying at home. A participant with a newborn stated, “My tension is related to my children. Like now, I have a newborn baby who is only 30 days old. I am fearful that he might get infected with it. I am keeping him home all the time” (M01).

Some of the participants became concerned about acquiring and passing on the infection to their other children. Consequently, they became highly anxious and vigilant of mild symptoms possibly indicative of COVID-19 infection. As illustrated by a participant, “These mild ailments [like headache and fever], which I previously used to ignore, now make me scared and worried” (M02). Another pregnant woman with four previous children reported experiencing increased anxiety and bodily symptoms along with feeling short-tempered with her children during the lockdown period. She stated, “My mood has been impacted a lot, I feel irritated and annoyed at my children…and I feel very anxious, I don’t know what has happened to my brain, sometimes I feel dizzy, sometimes feel like fainting” (M03).

A few participants found staying at home and having no guests to be peaceful over the lockdown period. A 34-year-old participant living in a nuclear family reported feeling calm while staying at home as she kept herself busy with helpful activities. She stated, “I do not go outside because of the fear (of getting infected), and I have no trouble staying at home. I offer my prayers and recite the Holy Quran” (M08). Another participant reported improvements in her relationship with extended family because of lack of contact between them, “Thank God, it has not impacted my health and well-being…we neither visit relatives nor do they come to our house, so there are no issues or arguments with anyone” (M16).

#### 3.2.2. Nowhere to Deliver the Baby

The participants who were pregnant were extremely concerned about the unavailability of gynecological services during the pandemic. A 20-year-old participant recalled feeling anxious knowing that she could not deliver at a government hospital due to hospitals being overcrowded with COVID-19 patients. She stated, “We checked again and again whether they [hospital staff] had started doing deliveries or not. My mother went there to inquire and was told that they were only attending to the COVID patients. Others were not allowed. It was devastating for me. All I was thinking was, where would I go for my delivery?” (M14). Another participant shared her experience of going to the hospital for her sister’s delivery and feeling traumatized from witnessing the ill-treatment her sister received: “My sister was experiencing labor pains and we went to the hospital. Not even a single doctor attended to her due to the overload of corona patients. She was in a lot of pain and no one was listening to us, nor were we allowed to go near her. It was a very difficult time for us. We came home after about 10 h, feeling extremely tired. My sister delivered her baby at home with the help of a ‘daei’ [traditional birth attendant]” (M14).

Some participants shared their experiences of being denied support at government hospitals because of the services being shut down in order to limit viral transmission. Having limited or no resources to acquire health services at a private hospital, they felt uncertain about their ability to access antenatal checkups or labor and delivery services. A woman who was in her fourth pregnancy described, “Because of the coronavirus, I have faced so many health issues since my third trimester has started, and I don’t know where to seek help from. I can’t go to the hospital for checkups and I have stopped taking my medicines. I was going to a government hospital for checkups since I can’t afford private treatment. God only knows what will happen when the time of the delivery comes” (M06).

#### 3.2.3. Not Making Ends Meet

Pregnant women who were facing financial problems, particularly due to loss of income during the lockdown, found themselves in a very difficult situation. In addition to their struggle to meet the basic needs of their families, they were burdened with expenses for medications and medical tests (for which they had to pay at government hospitals) and travel costs to visit the hospital. As described by a participant who was close to her delivery date, this resulted in much anxiety and uncertainty: “I am worried about my financial situation as my delivery is expected soon. We cannot afford to meet household expenses and medical expenses. If this lockdown will continue, I don’t know what will happen. My husband has no job and I cannot go to work in this condition. I am so uncertain about my future” (M06). Likewise, a participant with five children explained how she worried about bearing the expense of additional medications in case of a cesarean delivery: “As you know, we are women. We have children at home whom we have to feed and meet their needs. If I have an operation, where will I get the money from? There is no income due to corona” (M18).

A few participants reported how financial constraints impacted their relationships with their spouses and families. This would at times escalate to severe distress for some women. A 27-year-old participant recalled feeling hopeless and angry during her pregnancy towards her husband for not being able to provide for their family. She stated, “Sometimes I wondered why I was alive. I thought about whether I should die or go to Edhi Center [a women’s refuge]. Why am I living with a person who has no means of earning? My elder sister asked me about my diet. I was so furious that I started to think about killing myself and my husband. I thought my life was useless as I could not even provide the basic necessities for my daughter” (M12).

Some participants reported borrowing money and having the added pressure of being in debt. A participant married to a rickshaw driver with a nine-week-old baby expressed her misery stating, “The financial setback is the one that scares me the most. My husband is a rickshaw driver and he has been at home for the last three or four months. There has been no work, no business, and no food, so we moved to our village because of this financial crisis. We lived in the village for a whole month, and we are now heavily in debt” (M11).

#### 3.2.4. Children Losing out on Education

The majority of the participants with school-aged children were concerned about their children either falling behind in their studies or completely missing the academic year. A participant with four children stated, “My sons’ academic year is being wasted. They will be one year older but still in the same class. Their exams were held before lockdown and they were promoted to the next classes, but their school hasn’t informed us about the curriculum they are going to follow. They might lose their year” (M06). Some participants felt that the lockdown jeopardized their children’s daily routine and impacted them both physically and mentally. A mother of four stated, “The children’s routines have been disturbed, like not going to school and not properly doing homework and not listening…My worst fear is that my children’s future is at stake and their routine is disturbed” (M01). Another mother of two believed that staying at home was affecting her children mentally: “Yes, children’s studies have been affected. It’s been six or seven months and schools are closed. Children are annoyed about staying at home. They are feeling irritated and bored. They have drifted away from their studies and their minds are not focused anymore” (M11).

### 3.3. Anxieties Related to COVID-19

#### 3.3.1. Fear of Getting Infected and Infecting the Newborn

The majority of women were living in overcrowded conditions with extended families in one house. They were concerned about family members going out, getting infected, and passing the infection on to the rest of the family. A participant with a nine-week-old baby was worried about the infection being brought to her home, “I was stressed and feeling tense. If someone goes out, I become anxious wondering, ‘What if they get infected, and they bring corona home?’ It has affected us both emotionally and financially” (M11). The same participant recalled that when she was pregnant, she dreaded getting infected and passing it on to her baby: “During the last month of pregnancy, I was tense about what [would happen] if I get infected by it and then pass the infection on to my baby” (M11).

Another major concern was getting infected and consequently not being able to look after their newborn or other children. A participant with two children stated, “I used to hear that during pregnancy, there is a high chance of getting infected with coronavirus, and I was very frightened. All the time I was thinking ‘What if I become infected—then how can I take care of my two children?” (M15). A few participants expressed no fear, either because they believed that taking the right precautions would protect them or because they believed that only fate determined if they would get infected or not. A mother of two expressed her lack of fear, stating, “I am not worried about getting infected. I am going out freely but taking precautions like using sanitizers and a mask. I believe that death is from Allah. Without Allah’s will, I can’t die” (M12).

#### 3.3.2. Threat to Survival

The majority of the participants reported fears of not being able to cope with the pandemic due to their husbands’ loss of means to earn a living. With minimal savings and no other source of income, the pandemic became a threat to their survival. An expectant woman with four children expressed her fear, stating, “We are going through a tough time because of it. My husband has no job and our savings are not going to last long. They will run out soon, and then what? We have a humble background. We can’t tell anyone that we don’t have anything to eat at home. We can’t save anything [like food or money]. I think people will not die of coronavirus, but they will die of starvation if this lockdown continues” (M06). The majority of the participants were living in rental housing and were worried about losing their homes. A woman with a four-month-old baby described her dread of being asked to vacate the house as they were failing to pay the rent: “I worry because we have no way to pay the rent. How long will the owner of this house tolerate it? One day he will ask us to leave the house. Where will we go then?” (M07).

#### 3.3.3. Perception of Health Professionals as Damaging

In addition to anxiety about being turned away or treated badly, some participants expressed a complete lack of trust in healthcare providers. They believed that using government health services was a source of risk to themselves and their babies. This was based on a belief that the government was conspiring to show a high number of deaths in order to obtain international funding. A woman in her last month of pregnancy refused to go to a government hospital fearing that doctors would inject her newborn baby with the intention of killing him/her. She stated, “The hospital staff referred me to another hospital, but my husband could not make up his mind. He said he did not feel like going there. Obviously, it’s all because of the coronavirus. I came to know recently that at the Center Hospital, they inject small children and kill them. Now pregnant mothers don’t trust them. I have just made my card for tests [for delivering] at the RAZI hospital [a private hospital]” (M04). Another participant recalled feeling mistrustful when she was pregnant: “Before my delivery, when I visited the hospital, they told patients not to come there due to overburden of corona patients. I had no money at the time of my delivery. I was very worried. Everyone was stopping me from going there [to the government hospital], and they were saying that at government hospitals, they will kill me by giving me an injection” (M12).

In addition to the misconception about newborns or mothers being killed at the hospital, there were rumors that people infected with the coronavirus were being killed by health professionals. Some participants were wary of seeking medical care in the case that they were infected. A participant in her third trimester expressed her fears in seeking medical care for ailments by stating, “Now there remains no more trust in hospitals. It’s been many months, and people are dying. And if we have a minor cough, we have heard that they [the doctors] kill [patients] by giving injections. So now we don’t feel like going out. God knows best” (M04).

## 4. Discussion

This qualitative study explored the impact of the COVID-19 pandemic on women with preexisting anxiety symptoms during their pre- and postnatal period. The key findings showed that issues around COVID-19 increased perinatal anxiety and were related to financial problems, overburdened health facilities, and a lack of trust in health professionals. Women had fears for their own and their baby’s health and safety. The majority felt confined to their homes and anxious about getting infected and/or passing on the infection to their babies. 

The COVID-19 pandemic has had a huge economic impact on Pakistan [31]. Even prior to COVID-19, 24.3% of the population were living below the poverty line [32]. A recent survey examining the socioeconomic impact of COVID-19 in Pakistan reported that 78% of the respondents had financial uncertainty about their future, and 64% reported that their earnings decreased due to the pandemic [33]. During the pandemic, especially in lockdowns, daily wage earners were hit the hardest, and their families struggled to make ends meet. In addition, business failures and job losses left many with no means of earning money. In the same survey, 78% of the respondents reported increased household food shortages [28]. These effects of the economic crisis are reflected in our findings. As reported in other studies, poor economic status and unemployment are significant risk factors for anxiety and depression [34,35,36,37,38], particularly for anxiety among adults below 40 years of age [35,39,40], the age group of the majority of our participants (see Table 1 above).

The financial situation made most of the study participants reliant on free maternity services at government hospitals. However, during the peak of the pandemic, government hospitals were pushed to the brink of their allocated capacity [41], and the large influx of COVID-19 patients made the hospital staff feel overworked and overwhelmed [42], causing regular services to be reduced and patients seeking routine care to feel neglected. Health services in other low- and middle-income countries have experienced similar challenges leading to limited maternity, sexual, and reproductive healthcare [43,44,45]. Research has also suggested that difficulties in seeking professional medical help [46] and attitudes of hospital staff [47] contribute to anxiety during pregnancy.

During the interviews, many of the participants reported relying on social media and news to learn about COVID-19 and receive updates. In the majority of cases, it helped to raise their awareness about the signs and symptoms of the virus and take protective measures. However, frequent updates on the number of people infected or dying raised serious concerns in some of the participants, who felt worried, fearing the worst possible outcome. Similar findings were reported in a study aimed to evaluate vicarious traumatization among participants aiding with COVID-19 control in China [48]. 

COVID-19, being a novel virus, lends itself to conspiracy theories and misinformation [49], creating excessive fear and anxiety. Several studies have identified that, during the COVID-19 pandemic, frequent social media usage exposes its users to contradictory reports or misinformation, leading to confusion and increased anxiety [50]. In Pakistan, during the pandemic’s peak, there were rumors spread through social media about health professionals terminating their patients to show a high death rate due to COVID-19 or falsely declaring patients as suffering from the coronavirus [51]. 

A survey conducted in Pakistan in October 2020 showed that 55% of the respondents doubted the coronavirus was real and 46% agreed with conspiracy theories about it [52]. Views and misconceptions of the spread of COVID-19 and its treatment highlighted that the majority of the general population in Pakistan believed that the number of COVID-19 patients had been exaggerated or that doctors were “killing” patients in order to take advantage of the severity of the crisis to garner international attention and aid [53]. 

In our study, such misinformation led some participants to believe that health professionals at government hospitals might harm them or their babies. This put them in a dilemma of where and how to deliver their babies; their lack of trust in health professionals prevented them from accessing free government maternity services while financial constraints created a barrier to accessing private healthcare. Furthermore, Pakistan has one of the highest maternal and infant mortality ratios in South Asia [54]. For many women who were already fearing for their own and their babies’ lives [55], the lack of trust in health professionals and the inaccessibility of private maternity services exacerbated their fears. 

COVID-19 also impacted the day-to-day activities of most of the participants. Some participants isolated themselves for fear of getting infected or passing on the infection to their newborns or elderly members of their family. They were also worried about taking proper care of children if they became unwell. Fears of getting infected and passing the virus on to their loved ones during the peak of the pandemic have been reported in other studies [45,56]. The participants with children attending school were worried about their children losing an academic year, lagging behind in their studies, and losing focus. The psychological distress due to school closures has been highlighted in a number of studies as one of the significant effects of the COVID-19 lockdown on parents [57]. 

The pandemic lockdown has also resulted in the participants losing out on essential activities outside the household (such as attending antenatal checkups, shopping) as well as social activities (such as visiting family and friends, celebrating religious festivals, weddings, and funerals). Not being able to visit their maternal homes, which otherwise would have been an important source of support during pregnancy, and/or not being able to attend significant family events led many to feel unsupported and isolated. Feeling confined to the home and the loss of distractions resulted in feeling overwhelmed and worried, causing spikes in anxiety. Social distancing, isolation [58,59,60], and feelings of being trapped [61,62] have been reported in other studies as sources of increased anxiety and depression among pregnant women.

There are several study implications. Common mental disorders during pregnancy have a negative impact on postnatal depression as well as infant outcomes. COVID-19 appears to have contributed to symptoms of anxiety already experienced by pregnant women. More research is needed to explore if this additional anxiety related to COVID-19 leads to a higher prevalence of depression in the perinatal period. The study also highlights the need to adapt the existing or develop new psychosocial interventions for perinatal depression and anxiety so that they address issues specific to the COVID-19 pandemic, such as those identified in our study. Using peers and community health workers [63,64] to deliver targeted information could alleviate anxiety about visiting health centers and reduce unsubstantiated fears of health professionals. The role of technology in providing support to anxious mothers could be explored [65]. The perinatal period is critical for both maternal as well as child well-being, and efforts to address the women’s heightened anxiety during the COVID-19 pandemic are likely to have public health benefits.

### Strengths and Limitations

The data were collected from the participants with pre-existing symptoms of anxiety, which allowed for an in-depth analysis of the impact of COVID-19 on perinatal anxiety. The study applied a robust qualitative research methodology. The framework analysis allowed for a systematic, transparent, and thorough analysis of the data. The coders worked in pairs to analyze the data, which reduced bias in the analysis. To maintain objectivity, reflexivity was ensured through discussions during data collection and analysis. The participants were recruited from a tertiary care hospital with a large catchment area spread over seven urban and semi-urban districts; therefore, the sample was likely representative of a large segment of the Pakistani population.

A major limitation was that the study was carried out in a short period of only four months; therefore, the long-term consequences of COVID-19 could not be explored. Furthermore, the data were collected through interviews over the telephone, which did not allow for observation of nonverbal cues or body language. During some interviews, lack of privacy and distractions over the telephone could have minimized the richness of the data collected. Additionally, the participants were recruited from the intervention arm; hence, their views could have been influenced by receiving the intervention sessions. Moreover, the data were collected from patients of a single hospital primarily accessed by low-income patients, and therefore future studies need to consider collecting data from multiple government hospitals as well as from private hospitals across several different parts of Pakistan. Future studies may also consider conducting interviews with women from rural areas, from high-income households, and of advanced maternal ages as well as with women’s family members and health professionals to more fully understand the impact of COVID-19 on different groups.

## 5. Conclusions

Qualitative data from a tertiary care hospital in Pakistan showed that COVID-19 appears to have contributed to symptoms of anxiety in women already predisposed to anxiety in the prenatal period. The perinatal period is critical for both maternal as well as child well-being, and efforts to address the women’s heightened anxiety during the COVID-19 pandemic are likely to have public health benefits.

## Figures and Tables

**Table 1:** Participants’ characteristics (*n* = 27).

Age	Mean = 27 years (range: 18 to 36 years)
Employment status of the husband	Employed = 23 (85.1%), unemployed = 4 (14.8%)
Number of children	Mean = 2 (range: 1 to 5 children)
Education	Mean = 9 years (range: 0 to 16 years)
Family structure	Joint = 21 (77.7%), nuclear = 6 (22.2%)

## Data Availability

The data that support the findings of this study are not publicly available due to participant confidentiality restrictions but are available from the corresponding author upon reasonable request.
